# Differences in the phototaxis of pollen and nectar foraging honey bees are related to their octopamine brain titers

**DOI:** 10.3389/fphys.2014.00116

**Published:** 2014-03-28

**Authors:** Ricarda Scheiner, Anna Toteva, Tina Reim, Eirik Søvik, Andrew B. Barron

**Affiliations:** ^1^Department of Biochemistry and Biology, University of PotsdamPotsdam, Germany; ^2^Department of Biological Sciences, Macquarie UniversitySydney, NSW, Australia

**Keywords:** biogenic amines, tyramine, division of labor, honey bee, light responsiveness, insect, behavior

## Abstract

The biogenic amine octopamine is an important neuromodulator, neurohormone and neurotransmitter in insects. We here investigate the role of octopamine signaling in honey bee phototaxis. Our results show that groups of bees differ naturally in their phototaxis. Pollen forgers display a lower light responsiveness than nectar foragers. The lower phototaxis of pollen foragers coincides with higher octopamine titers in the optic lobes but is independent of octopamine receptor gene expression. Increasing octopamine brain titers reduces responsiveness to light, while tyramine application enhances phototaxis. These findings suggest an involvement of octopamine signaling in honey bee phototaxis and possibly division of labor, which is hypothesized to be based on individual differences in sensory responsiveness.

## Introduction

The biogenic amine octopamine is a pivotal insect neurotransmitter, neurohormone and neuromodulator (Evans, 1980; Roeder, 1999; Blenau and Baumann, 2003; Scheiner et al., 2006). It has many and diverse physiological functions including the modulation of complex behaviors such as aggression in crickets (Stevenson et al., 2005) or learning and memory in honey bees (Behrends and Scheiner, 2012). The majority of studies on octopamine investigate the action of this transmitter on peripheral targets such as muscles, because they are easily accessible to experimental manipulation (for review see Roeder, 2005). In this paper, we concentrate on the role of octopamine in the central nervous system of honey bees.

Octopamine often has an arousing effect. In locusts, for example, application of octopamine can dishabituate the habituated response of descending movement detector interneurons to repetitive visual stimuli (Bacon et al., 1995). In honey bees, octopamine enhances responsiveness to gustatory stimuli (Scheiner et al., 2002), improves appetitive learning (Behrends and Scheiner, 2012) and increases the ability of bees to discriminate nestmates from non-nestmates (Robinson et al., 1999). We here asked if octopamine would also have an enhancing effect on another stimulus modality, i. e., responsiveness to light. As the highest concentration of octopamine receptors in the brain can be found in the optic lobes (Roeder and Nathanson, 1993), it can be assumed that octopamine has important modulatory functions in the visual system of honey bees.

To study the function of octopamine in phototaxis, we were looking for groups of bees which naturally differ in this behavior. Honey bees display a complex division of labor. Among the group of foragers, for example, some bees collect pollen, while others collect nectar (Winston, 1987; Seeley, 1995). These bees further differ in physiological and behavioral aspects. Pollen foragers, for example, are more responsive to gustatory stimuli than nectar foragers (Page et al., 1998; Scheiner et al., 1999, 2001, 2003). For that reason, they perform better in appetitive learning assays than nectar foragers (Scheiner et al., 1999, 2001). It was earlier hypothesized that responsiveness to light and responsiveness to gustatory stimuli would be regulated jointly in the central nervous system of the bee (Erber et al., 2006). We therefore hypothesized that pollen and nectar foragers would naturally differ in their phototaxis. Finding indeed a systematic difference in the phototaxis of pollen and nectar foragers, we were looking for molecular correlates of these behavioral differences with respect to octopamine signaling. Our focus was on brain neuropils involved in visual processing. On the level of gene expression we compared the expression of two octopamine receptor genes between pollen and nectar foragers. The honey bee possesses five octopamine receptors (Hauser et al., 2006; Balfanz et al., in press). One of them, *AmoctαR1*, has been studied in some detail by different groups (Farooqui et al., 2003; Grohmann et al., 2003; Beggs et al., 2011; Sinakevitch et al., 2011, 2013). It is linked to a Ca^2+^ signaling cascade (Grohmann et al., 2003). The other four receptors are linked to the cAMP signaling cascade and have only recently been characterized (Balfanz et al., in press). We selected the only Ca^2+^ linked octopamine receptor (*AmoctαR1*) and one representative of the cAMP-coupled octopamine receptors (*AmoctβR4*) for our studies. At the level of octopamine signaling, we compared intrinsic octopamine titers between pollen and nectar foragers. Finally, we tested if elevation of octopamine titers would directly affect phototaxis.

## Materials and methods

### Collection of bees

To measure phototaxis and locomotion of honey bee foragers, bees from a colony were collected on their return from a foraging trip. Pollen foragers were recognized by their large pollen loads, since these bees usually do not collect any additional nectar. Returning bees with extended abdomens and without any pollen on their hind legs were regarded as nectar foragers, although a minority of them may have been water collectors (Scheiner et al., 2013). The small number of bees returning with both nectar and a small pollen load were not used for this study. After collecting bees, they were immobilized by chilling on ice and were subsequently mounted in small brass tubes as described in Scheiner et al. (2013). Bees in the group “returning foragers” were only fed with 5 μl of a 30% sucrose solution to prevent starvation, particularly in the group of pollen foragers, which usually return from a foraging trip with an empty honey stomach. Bees in the group “satiated foragers” were fed to repletion with 50% sucrose, i.e., until they did not show the proboscis extension response to a 50% sucrose solution. Bees rested for 1 h after mounting.

Returning nectar foragers collected for behavioral pharmacology were directly placed into a feeding cage. After a 3-day treatment with either a 50% sucrose solution or a biogenic amine dissolved in a 50% sucrose solution bees were captured individually from the cage.

### Measuring locomotion and phototaxis

Before bees were released into the dark phototaxis arena, they were individually placed in a Petri dish of 10 cm diameter which had several three-millimeter-openings in the top lid to allow air influx. Bees rested in a dark room, which was lit by a dim red light, for about 10–20 min before they were released into the arena. Here, each bee was first tested for its locomotion and the walking path of the bee in total darkness was randomly recorded for 30 s out of a 90-s period without visual stimulation (Erber et al., 2006; Scheiner et al., 2013). Phototaxis was measured as in Erber et al. (2006). Basically, a bee was placed in the dark arena and positive phototaxis was elicited by turning on one of twelve green light emitting diodes (520 nm). The light sources were fixed in 30° steps around the perimeter of the 35-cm arena. Light sources were fit to neutral density filters to attenuate light intensity. The following logarithmic order of relative light intensities was used: 100, 50, 25, 12.5, 6.25, and 3.125%. Two diodes with the same relative intensity were always mounted opposite each other. Once the bee had reached the light source, the diode was turned off and the same light intensity on the opposite side of the arena was switched on. This procedure was repeated four times for each light intensity. The walking time a bee needed to reach a certain light source was taken by a stop watch. For comparisons, we calculated the mean walking time of a bee toward one light intensity.

### Behavioral pharmacology

For behavioral pharmacology, bees were allowed to feed *ad libitum* on sugar water (30%; 0.9 mol/l) containing octopamine, tyramine or no amine for 3 days. This application method has been used successfully to enhance titers of biogenic amines in the brain of honey bees (Schulz and Robinson, 2001; Barron et al., 2007). Other methods to increase octopamine brain titers, for example by local injection, were not applicable for the duration of treatment.

The following treatments were applied:

1. 30% sucrose
2. 30% sucrose + octopamine (10^−3^ mol/l)
3. 30% sucrose + octopamine (10^−2^ mol/l)
4. 30% sucrose + tyramine (10^−3^ mol/l)
5. 30% sucrose + tyramine (10^−2^ mol/l).

### Quantitative real-time PCR

For quantification of octopamine receptor gene expression, brains of bees were dissected in ice-cold bee saline (NaCl 270 mM, KCl 3.2 mM, MgCl_2_10 mM, CaCl_2_1.2 mM, 3-(N-morpholino)propanesulfonic acid (MOPS) 10 mM, pH 7.3) directly after measuring locomotion and phototaxis. After removal of the trachea, hypopharyngeal glands, salivary glands, retinal pigment, antennal lobes, and suboesophageal ganglion, the optic lobes and the mushroom bodies were separated and immediately frozen in liquid nitrogen. RNA extraction and cDNA synthesis were performed as in Reim et al. (2013). In addition, an on-column DNase digestion step was introduced in RNA extraction. After binding of the RNA to the membrane of the column, samples were incubated with 30 Kunitz units DNase (Qiagen, Hilden, Germany) for 15 min at room temperature. For cDNA synthesis about 100 ng total RNA was used.

The qPCR analysis was performed on a Rotor Gene Q (Qiagen, Hilden, Germany). The sequence specific TaqMan probes had a BlackBerry Quencher (BBQ) on the 3′end and a fluorophore on the 5′end. For the receptors we used Hexachlorfluorescein (HEX), the reference gene *ef1α* was fused to Fluorescein (FAM). Sequences of primers and probes used for gene-specific amplification are given in Table 1. Brain parts of each bee were analyzed separately. Two cDNA duplicates were used from each single tissue sample and each cDNA duplicate was tested in triplicates. The chemicals, the protocol and the quantification analysis we used followed the instructions in Reim et al. (2013). In the present study we used different standard concentrations for calculating the amount of copies in the samples. Four increasing quantities of DNA (10^3^ –10^6^ copies per reaction) of the respective gene were used. Expression of octopamine receptor mRNA was calculated relative to the reference gene *ef1α*, which did not differ in expression between pollen and nectar foragers (Reim et al., 2013). For graphic display, pollen foragers were set to one.

**Table 1:** **Accession numbers (EMBL) of the analyzed genes and their sequences of primers and probes used for qPCR assay**.

**Gene**	**Accession number**	**Primers and probes (5′→3′)**
*ef1α*	AY721716	Forward: GAACATTTCTGTGAAAGAGTTGAGGC
		Reverse: TTTAAAGGTGACACTCTTAATGACGC
		Probe: ACCGAGGAGAATCCGAAGAGCATCAA
*AmoctαR1*	AJ547798	Forward: GCAGGAGGAACAGCTGCGAG
		Reverse: GCCGCCTTCGTCTCCATTCG
		Probe: TCCCCATCTTCATCACCCTTGGCTTCTCC
*AmoctβR4*	HF548212	Forward: CACTTCGATACGACAACAAACG
		Reverse: GGTTCAGGGCGCTGTTGA
		Probe: ACCACGTCCTTGTGCGGCGA

### High-performance-liquid-chromatography

For high-performance-liquid-chromatography (HPLC), the head of a pollen or nectar forager was removed and stored in small 1.5 ml reaction tubes at −80°C until use. Before dissecting optic lobes and mushroom bodies the heads of the bees were freeze-dried at −65°C and 320 mTorr for 45 min (Virtis benchtop freeze drier model no. 2KBTXL-75). Afterwards, the brains were dissected on dry ice to prevent defrosting. The optic lobes and the mushroom bodies were separated. The antennal lobes and the suboesophageal ganglion were removed.

Biogenic amine levels were measured using HPLC coupled to a coulometric electrochemical detector (Søvik et al., 2013). To extract biogenic amines, brain regions were centrifuged at 15 G for 5 minutes in a refrigerated centrifuge cooled to 4°C, and then homogenised by ultra-sonication in 20 μl of 0.2 M perchloric acid containing 10 pg/μl of the HPLC standard dihydroxybenzylamine. Samples were then incubated for 20 min at 0°C protected from light. Post incubation samples were centrifuged at 15 G and 4°C for 15 min to pellet cellular debris. Thirteen μl of the supernatant of each sample was introduced to an Agilent 1200 Series HPLC system with an HR-80 column with 0.2 micron octadecylsilane packing for sample separation. Biogenic amine content was quantified using an ESA Coulochem III electrochemical detector using an ESA 5011A high-sensitivity dual-electrode analytical cell (Agilent Technologies, Santa Clara, CA). Amines were quantified on Channel B operating at 800 mV. Amounts of octopamine were quantified relative to known amounts of this chemical as standard, and relative to DHBA as the internal standard. All chemicals were supplied by Sigma-Aldrich (St. Louis, MO, USA). See Søvik et al. (2013) for a more detailed description of the HPLC method utilised here.

### Statistics

Responsiveness to the different light intensities was compared between pollen and nectar foragers of different satiation levels or between different treatments using repeated-measurement analysis of variance (ANOVA, SPSS 21) on the mean walking times of each bee to each of the six different light intensities. Walking speed in the dark arena was compared using ANOVA with Tukey Kramer post hoc tests. Walking distance in the dark arena was measured using a computer algorithm (Erber et al., 2006). Mean relative expression was calculated and compared between pollen and nectar foragers using two-tailed *T* tests. Similarly, titers of octopamine in the optic lobes and mushroom bodies were compared between pollen and nectar foragers using two-tailed *T* tests.

## Results

**Figure 1:**
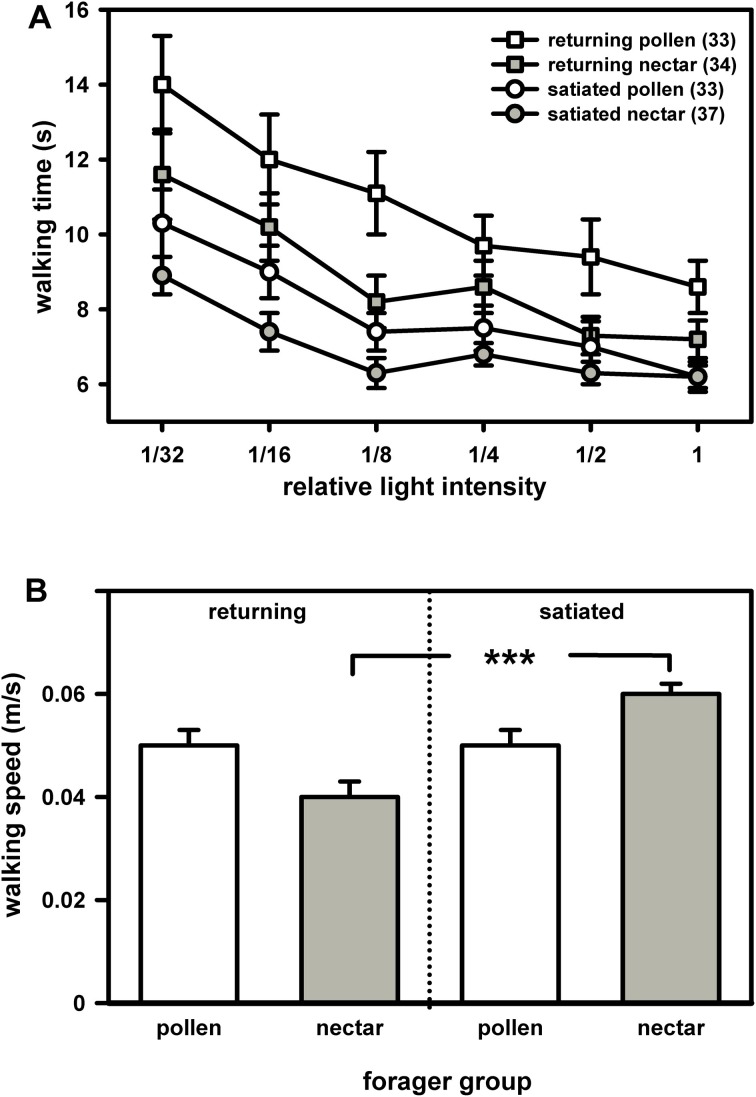
**Phototaxis (A) and locomotion (B) of returning and of satiated pollen and nectar foragers. (A)** Bees generally preferred light sources of higher intensity over those of lower intensity and consequently walked faster towards the former. Feeding generally reduced walking times towards the different light sources. Nevertheless, nectar foragers went faster to the light than pollen foragers, regardless of whether they had just returned from a foraging trip or had been satiated prior to testing. For statistics see text. **(B)** Pollen and nectar foragers did not differ in their walking speed in the dark arena, regardless of whether they had just returned from a foraging trip or whether they had been satiated prior to testing. Feeding significantly increased walking speed in nectar foragers but had no effect on walking speed in pollen foragers. The significant difference is indicated by asterisks (^***^: *P* ≤ 0.001, Tukey Kramer post hoc test). Both figure parts display mean values and standard errors. The number of bees tested is indicated in brackets behind each group.

We compared the phototaxis, i.e. the walking times the bees needed to reach six different light intensities, between returning pollen and nectar foragers of the honey bee. Because returning pollen foragers generally display a lower degree of satiation than returning nectar foragers, we also compared the phototaxis of pollen and nectar foragers which had been fed to satiation prior to the phototaxis test (Figure 1).

Generally, foragers preferred high light intensities over low light intensities and needed significantly less time to reach the higher light intensities (Figure 1A: *F*_(5, 132)_ = 6.58, *P* = 0.001, ANOVA, effect of light intensity). This preference was similar in pollen and nectar foragers. There was no interaction between light intensity and forager type [*F*_(5, 132)_ = 0.89, *P* > 0.05, ANOVA, interaction effect light intensity × foraging role] or between light intensity and the degree of satiation [*F*_(5, 132)_ = 0.59, *P* > 0.05, ANOVA, interaction effect of light intensity and satiation].

Intriguingly, pollen foragers spent more time walking towards most of the light intensities than nectar foragers (Figure 1A; *F*_(1, 132)_ = 8.52, *P* = 0.01, ANOVA, effect of foraging role). This behavioral difference was independent of their locomotor behavior in the dark arena [*F*_(1, 132)_ = 0.49, *P* > 0.05, ANOVA, effect of walking speed in the dark]. The degree of satiation strongly affected phototaxis [Figure 1A; *F*_(1, 132)_ = 17.18, *P* = 0.001; ANOVA, factor satiation]. Satiated foragers walked significantly faster toward the light than did returning foragers. But satiated nectar foragers still walked significantly faster towards the different light intensities than satiated pollen foragers. There was no interaction between foraging role and satiation with respect to phototaxis [*F*_(1, 132)_ = 1.18, *P* > 0.05, ANOVA, interaction effect of foraging role and satiation].

Foraging role did not affect locomotion in the dark [Figure 1B; *F*_(1, 136)_ = 0.30, *P* > 0.05; ANOVA, factor foraging role]. If the walking speed in the dark arena is indicative of the walking speed of the bees in the light (which was not measured in our assay), our data suggest that pollen foragers walked less directly to the different light sources, since they did not differ from nectar foragers in their walking speed *per se*.

Satiation significantly affected walking speed in the dark arena [*F*_(1, 136)_ = 22.23, *P* = 0.001; ANOVA, factor satiation]. Satiated foragers, particularly nectar foragers, walked significantly faster in the dark arena than did returning foragers [*F*_(1, 136)_ = 4.45, *P* = 0.05; ANOVA, interaction effect of satiation x foraging role].

**Figure 2:**
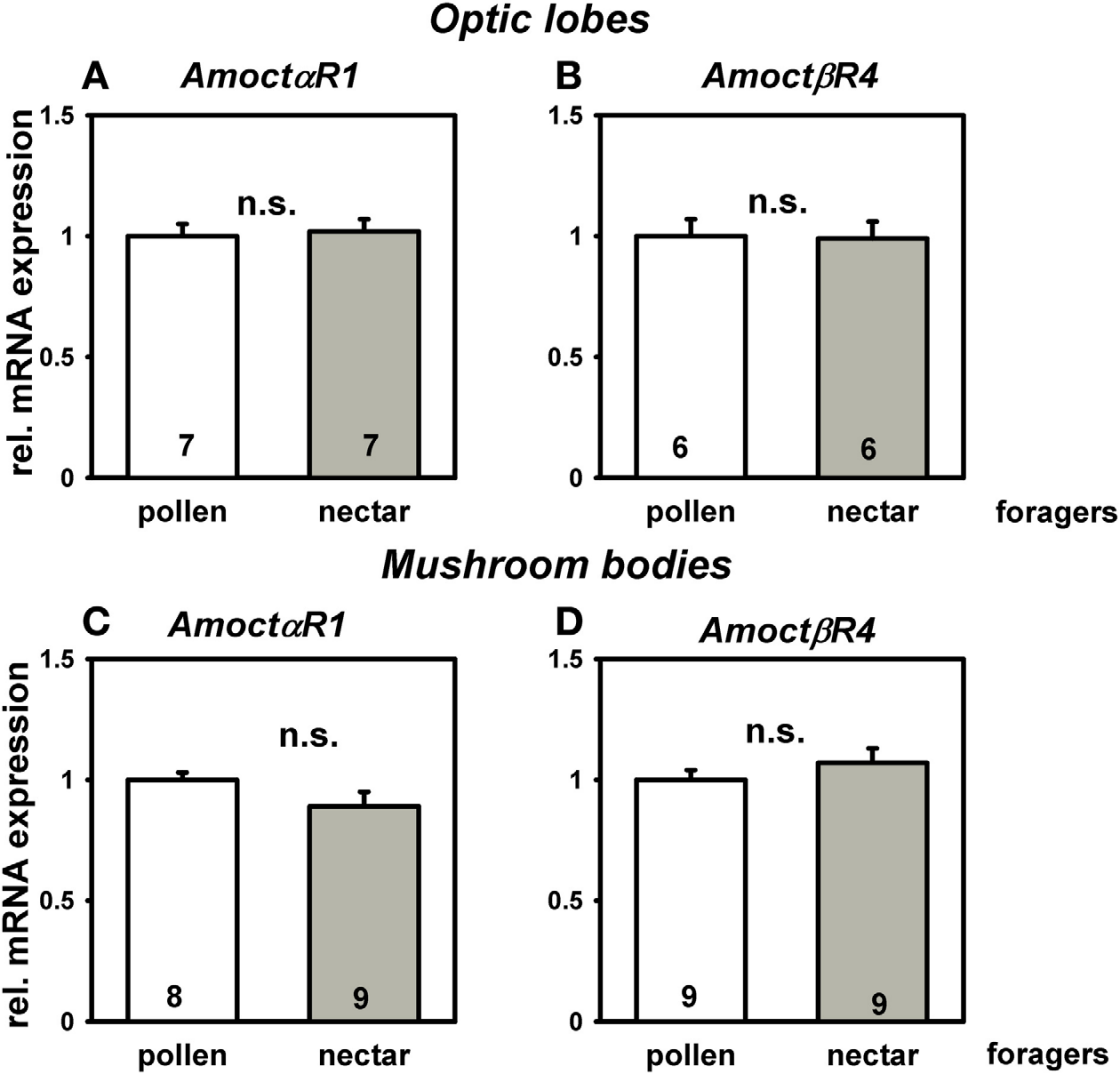
**Relative messenger RNA expression of the honey bee octopamine receptor genes *AmoctαR1* (A,C), and *AmoctβR4* (B,D) in brain neuropils important for visual processing of pollen and nectar foragers, i.e., optic lobes (A,B) and mushroom bodies (C,D)**. Mean expression relative to the reference gene *ef1α* and standard errors are displayed. Pollen foragers were set to one. Number of bees tested is given for each group. Pollen and nectar foragers do not differ in their mRNA expression of the measured octopamine receptor genes in major brain neuropils involved in visual processing (*P* > 0.05, *T* test). Groups not differing from each other are marked with “n.s”.

We next asked if the higher responsiveness to light of nectar foragers was related to a different octopamine receptor gene expression in brain neuropils involved in visual processing, i.e. optic lobes and mushroom bodies. The gene *AmoctαR1* codes for Ca^2+^-coupled octopamine receptor (Grohmann et al., 2003). The gene *AmoctβR4* codes for a cAMP-linked octopamine receptor (Balfanz et al., in press). Expression of the octopamine receptor genes *AmoctαR1* and *AmoctβR4* did not differ between pollen and nectar foragers in both brain neuropils involved in visual processing (Figure 2; *P* > 0.05 *T* test). This suggests that the differences in sensory responsiveness of pollen and nectar foragers are not causally linked to differences in octopamine receptor gene expression.

**Figure 3:**
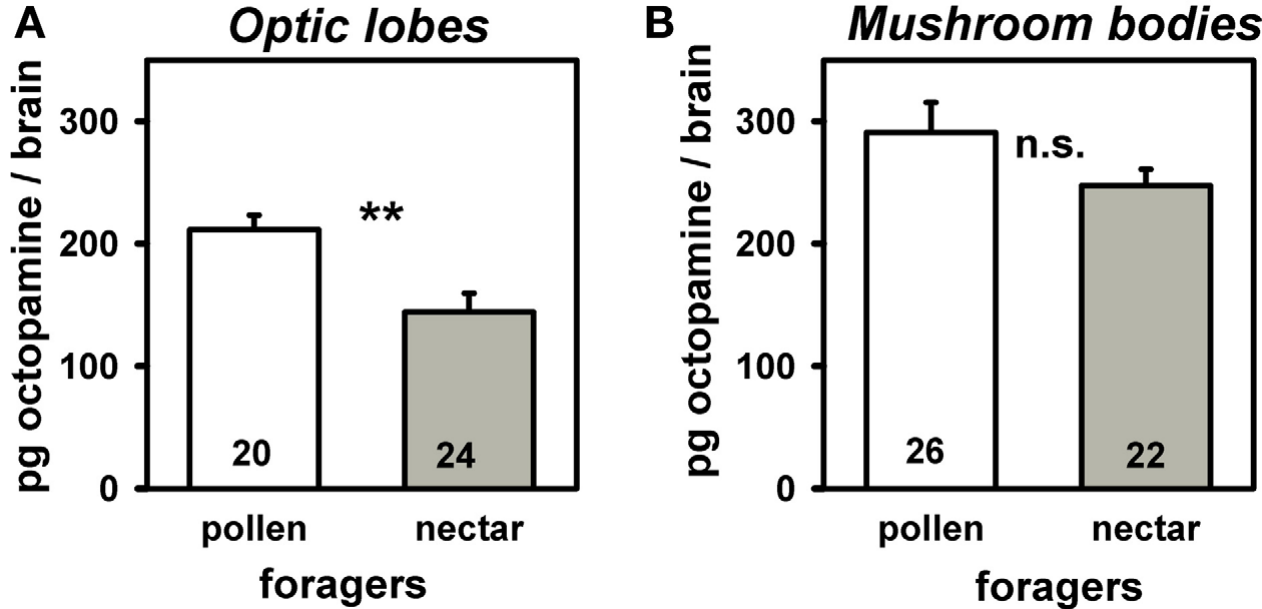
**Amount of octopamine in optic lobes (A) and mushroom bodies (B) of pollen and nectar foragers**. Mean titers and standard errors are displayed. Number of bees tested is given for each group. Pollen foragers had significantly more octopamine in their optic lobes than nectar foragers (^**^: *P* ≤ 0 0.01, *T* test). The other groups did not differ significantly from each other and are marked with “n.s.”

In a further experiment we investigated if octopamine titers in the two brain neuropils associated with visual processing (i.e., optic lobes and mushroom bodies) differ between pollen and nectar foragers. The octopamine titer in the optic lobes of pollen foragers was significantly higher than that of nectar foragers (Figure 3A; *T* = 3.34, *n*_pollen_ = 20, *n*_nectar_ = 24, *P* = 0.01), while octopamine titers did not differ between pollen and nectar foragers in the mushroom bodies (Figure 3B: *T* = 1.56, *n*_pollen_ = 26, *n*_nectar_ = 22, *P* > 0.05). This suggests that the reduced attraction to light observed in pollen foragers might be linked to their higher octopamine titer in the optic lobes. If this were the case, we hypothesized that increasing octopamine brain titers should reduce responsiveness to light and therefore increase walking times towards light. To test this hypothesis, we treated foragers orally with octopamine and subsequently analyzed their phototaxis and locomotion.

**Figure 4:**
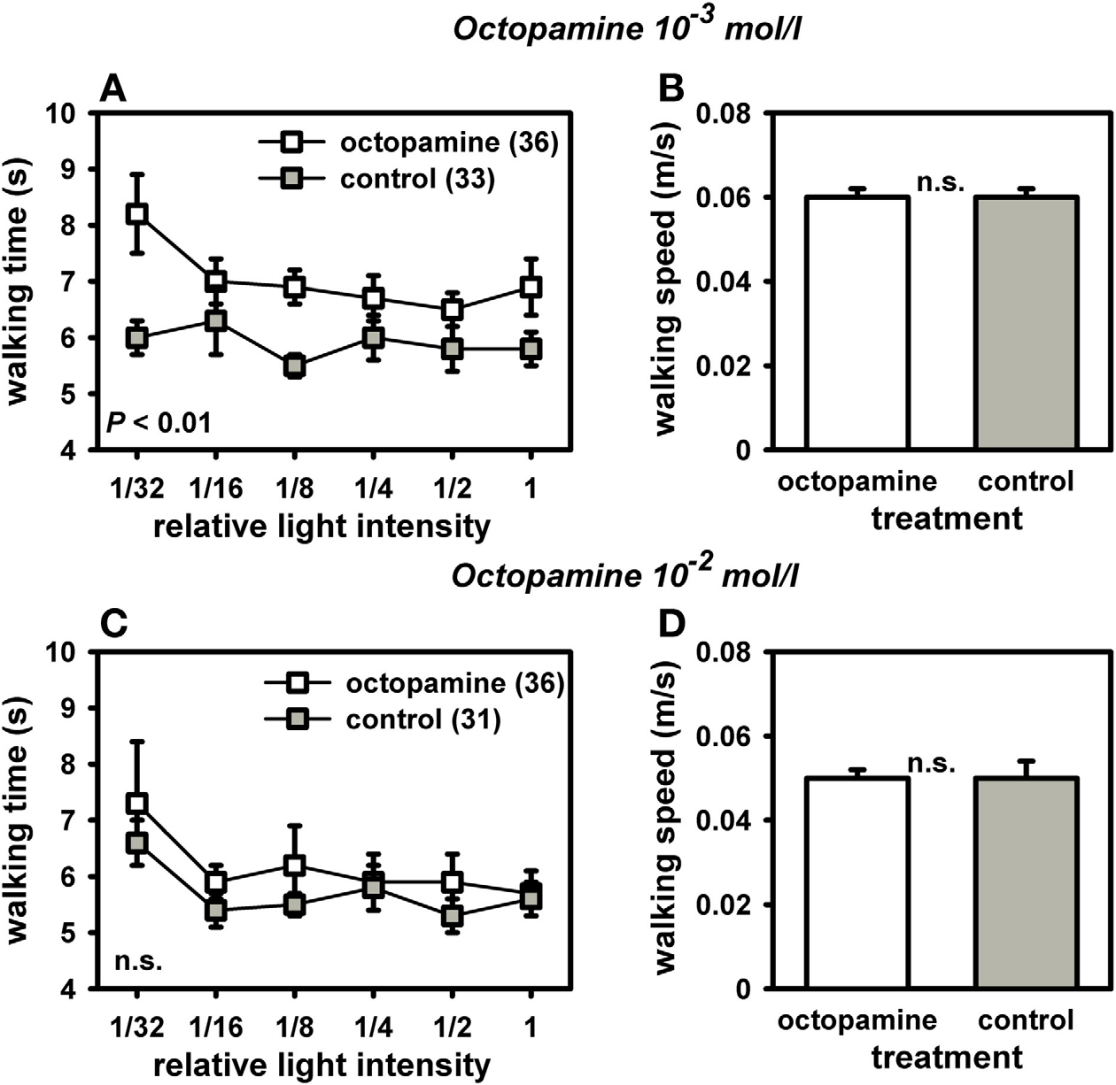
**Phototaxis and locomotion of nectar foragers treated with octopamine 10^−3^ mol/l or octopamine 10^−2^ mol/l. For phototaxis (A,C), mean walking time towards the different light intensities and standard errors are shown**. For locomotion **(B,D)**, mean walking speed in the dark arena and standard errors are given. **(A)** Bees treated with octopamine (10^−3^ mol/l) walked significantly more slowly to the different light sources than control bees treated with sucrose (*P* ≤ 0.01, ANOVA, effect of treatment). **(B)** This difference in phototaxis was independent of their locomotion in the dark arena, which did not differ between groups (*P* > 0.05, ANOVA, effect of walking speed in the dark). **(C)** Bees treated with octopamine (10^−2^ mol/l) did not differ in their phototaxis from controls treated with sucrose (*P* > 0.05, ANOVA, effect of treatment). **(D)** Locomotion in the dark arena also did not differ between the two groups (*P* > 0.05, ANOVA, effect of walking speed in the dark). The number of bees tested is indicated in brackets behind each group. Groups not differing from each other are marked with “n.s.”

Octopamine-treated foragers walked significantly more slowly toward light and thus displayed a reduced light responsiveness compared to controls (Figure 4A). The octopamine effect was dose-dependent with octopamine in the concentration of 10^−3^ mol/l yielding a significant effect [Figure 4A; *F*_(1, 68)_ = 6.76, *P* = 0.01; ANOVA effect of treatment), while octopamine 10^−2^mol/l had no significant effect on light responsiveness [Figure 4C; *F*_(1, 67)_ = 1.67, *P* > 0.05; ANOVA effect of treatment]. Neither treatment affected walking speed in the dark arena [10^−3^ mol/l: Figure 4B; *F*_(1, 68)_ = 1.59, *P* > 0.05; ANOVA effect of walking speed in the dark; 10^−2^ mol/l: Figure 4D: *F*_(1, 67)_ = 2.08, *P* > 0.05; ANOVA effect of walking speed in the dark]. If the walking speed of the bees in the dark arena is indicative of their walking speed in the light, our findings suggests that the slower walking speed of the octopamine-treated bees toward the lights is mostly related to their different evaluation of the light sources and not to a generally reduced locomotion.

**Figure 5:**
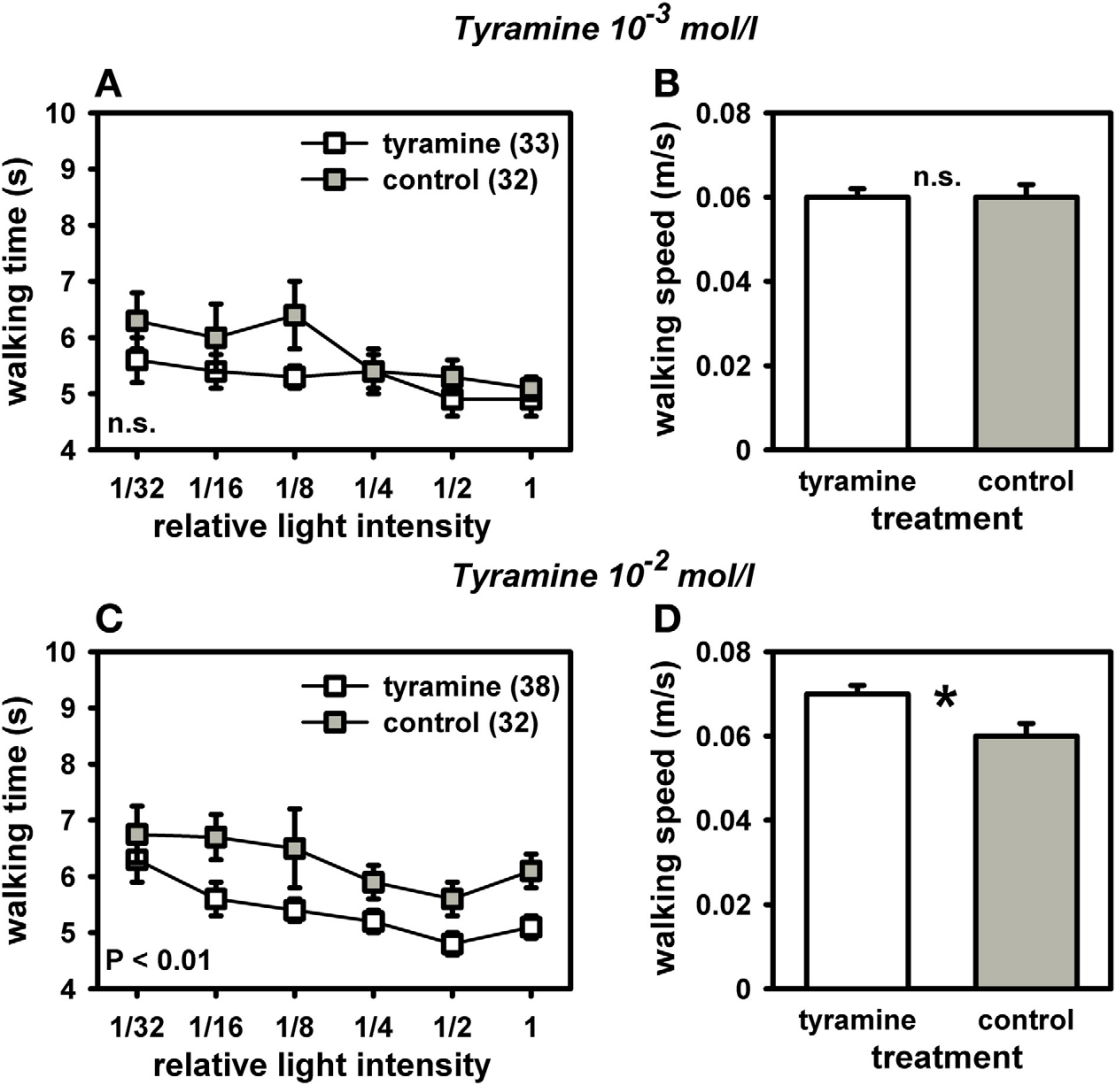
**Phototaxis and locomotion of nectar foragers treated with tyramine 10^−3^ mol/l or tyramine 10^−2^ mol/l. Details as in Figure 4. (A)** Bees treated with tyramine (10^−3^ mol/l) did not differ in their phototaxis from control bees treated with sucrose (*P* > 0.05, ANOVA, effect of treatment). **(B)** These two groups also did not differ in their walking speed in the dark arena (*P* > 0.05, ANOVA, effect of walking speed in the dark). **(C)** Bees treated with tyramine (10^−2^ mol/l) walked significantly faster to the different light sources than control bees treated with sucrose (*P* ≤ 0.01, ANOVA, effect of treatment). **(D)** Bees treated with tyramine (10^−2^ mol/l) also walked significantly faster than controls in the dark arena (*P* ≤ 0.05, ANOVA, effect of walking speed in the dark), indicating an effect of higher walking speed in the dark on phototaxis. Significant differences between groups are indicated in the Figure or by asterisks (^*^: *P* ≤ 0.05, ANOVA). Groups not differing from each other are marked with “n.s.”

Because octopamine in high concentrations can also bind to a honey bee tyramine receptor (Blenau et al., 2000), we treated another set of bees with tyramine to test for specificity of our octopamine-effect. Intriguingly, tyramine-treated bees walked significantly faster toward the light and had a higher walking speed in the dark arena (Figure 5). This effect was dose-dependent. Tyramine in the concentration of 10^−2^ mol/l significantly reduced walking times to the light [Figure 5C: *F*_(1, 71)_ = 6.81, *P* = 0.01; ANOVA, effect of treatment]. This tyramine concentration also increased walking speed in the dark arena [Figure 5D; *F*_(1, 71)_ = 6.21, *P* = 0.05; ANOVA, effect of walking speed in the dark], demonstrating an effect of tyramine on locomotor behavior. It can be assumed that the faster walking times to the light sources induced by the tyramine treatment were at least partially a result of the tyramine effect on locomotion. In contrast, tyramine in the concentration of 10^−3^ mol/l did not affect walking times towards the light [Figure 5A; *F*_(1, 71)_ = 1.40, *P* > 0.05; ANOVA, effect of treatment]. It had no effect on walking speed in the dark arena either [Figure 5B; *F*_(1, 71)_ = 1.81, *P* > 0.05; ANOVA, effect of walking speed in the dark]. These findings suggest that octopamine specifically reduces responsiveness to light without affecting locomotor behavior, while tyramine increases phototaxis, most likely through enhancing locomotion.

## Discussion

### Octopamine affects phototaxis in honey bee foragers

Octopamine frequently has arousing functions in the insect nervous system. We therefore hypothesized that it would increase phototaxis and high octopamine titers would correlate with higher responsiveness to light. To our surprise, this transmitter had the opposite effect on honey bee phototaxis. Pollen foragers, which naturally have higher octopamine titers in the optic lobes than nectar foragers without differing in their octopamine receptor expression, displayed a significantly reduced phototaxis. Systemically elevating octopamine titers reduced phototaxis, while elevating tyramine titers increased responsiveness to light. These data suggest that octopamine specifically modulates phototaxis, with high octopamine titers inhibiting responsiveness to light. Interestingly, our data are supported by an earlier study on phototaxis in fruit flies. In that species, too, activating octopamine receptors through the octopamine receptor agonist chlordimephorm led to a reduced phototaxis (Dudai et al., 1987).

We assume that our octopamine-induced effects can be attributed to activation of specific octopamine receptors in the honey bee brain. All five octopamine receptors from the honey bee have now been cloned and characterized (Grohmann et al., 2003; Balfanz et al., in press). While four of these receptors increase intracellular cAMP levels upon activation, one receptor is coupled to Ca^2+^. Experiments in *Drosophila melanogaster* showed that increasing cAMP levels directly or indirectly by applying octopamine slowed down the kinetics of light response (Chyb et al., 1999). It is conceivable that the octopamine application in our experiments had similar effects, possibly activating via one or more of the cAMP-coupled octopamine receptors in the optic lobes and ultimately leading to a lower walking speed toward the different light intensities. Activation of the tyramine receptor AmTYR1, in contrast, reduces cAMP levels (Blenau et al., 2000) and should therefore have the opposite effect on phototaxis. Our experiments demonstrate that a systemic increase in tyramine titers indeed enhances responsiveness to light. Tyramine is generally believed to act antagonistically to octopamine (Roeder, 2005). Our results provide experimental evidence for this hypothesis with respect to responsiveness to light. However, tyramine also increased walking speed in the dark arena in our experiments. This suggests that the increased phototaxis induced by tyramine treatment was, at least partially, caused by the tyramine effect on locomotion. Octopamine, in contrast, had no effect on locomotion. Further support for the hypothesis that tyramine and octopamine act antagonistically in the nervous system comes from experiments on honey bee flight (Fussnecker et al., 2006), and on the initiation of foraging behavior (Schulz and Robinson, 2001). Taken together, these data suggest an important antagonistic regulatory function of octopamine and tyramine in honey bee behavior.

Clearly, more experiments are needed to specify the role of octopamine in honey bee vision and light responsiveness. With the approach of RNA interference techniques in the honey bee (Farooqui et al., 2003), it will soon be possible to relate the octopamine effect to individual octopamine receptors. In addition, it will be helpful to produce specific antibodies against individual octopamine receptors to study their localization throughout the brain. Methods like RNA interference and determination of mRNA expression should also enable us to evaluate the activity of enzymes involved in octopamine synthesis, such as tyramine-β-hydroxylase. Future experiments can then elucidate the role of octopamine synthesis in modulating sensory responsiveness.

### Limitations of the method

Oral application of octopamine is an effective non-invasive method to chronically increase octopamine brain levels (Schulz and Robinson, 2001) and to induce behavioral changes, as shown by our experiments. However, the mechanisms which control the observed changes in behavior are unclear. Although we found a relationship between high octopamine titers in the optic lobes and lower responsiveness to light, our method of octopamine application has the disadvantage of targeting not only the optic lobes but also peripheral tissues and other neuropils in the brain. We therefore cannot exclude the possibility that the applied octopamine acted through peripheral sensory organs to reduce light responsiveness. However, octopamine seems to increase peripheral responses to light rather than to reduce them, as indicated by electroretinogram recordings (Franz, 2007). Therefore, we assume that our behavioral changes are due to processes within the brain. We assume that higher octopamine concentrations in the optic lobes trigger the evaluation of light stimuli perceived through the eyes and modulate behavioral responses respectively.

For gustatory inputs it was shown that oral octopamine application leads to increased proboscis responses to low-concentrated sucrose solutions which the antennae could perceive (Scheiner et al., 2002). The changed evaluation of gustatory stimuli within the brain led to an improved associative learning performance (Behrends and Scheiner, 2012), most likely through simulating higher sucrose rewards, because high subjective sucrose rewards correlate with better learning performance (Scheiner et al., 1999, 2005). Similarly, octopamine application might lead to a reduced evaluation of light stimuli, leading to reduced phototaxis. Admittedly, injections of octopamine could have been performed more locally. But an injection only works effectively up to 2 h (Linn et al., 1994; Scheiner et al., 2002). For long-term treatment, bees would have to be injected multiple times, which would be too stressful, since each injection causes stress (Harris and Woodring, 1992). Oral administration of octopamine has the great advantage of inducing low levels of stress, if inducing any stress at all, so that behavioral changes observed after treatment are more likely to result from the administered substance or its metabolic products than from stress effects caused by the treatment.

### Pollen foragers are less responsive to light than nectar foragers

Nectar foragers went faster to the different light intensities than pollen foragers, independent of their locomotor behavior in the dark arena. The difference in phototaxis between the two groups of bees was also independent of their degree of satiation, since returning and satiated pollen and nectar foragers differed in their phototaxis. Our experiments thus provide direct evidence for the notion that honey bee division of labor is based upon or correlates with individual differences in sensory response thresholds (Robinson, 1992; for review see Beshers et al., 1999).

The differences in phototaxis between pollen and nectar foragers only partly support results of Tsuruda and Page (2009) who found that pollen foragers walked slightly faster to the lowest light intensity in a similar phototaxis assay but did not differ from nectar foragers in their walking time to higher light intensities. However, the two assays differ considerably. In particular, the arena of Tsuruda and Page (2009) had a smaller diameter of 25 cm, compared to our arena (diameter: 35 cm), which may explain the relatively faster walking times in their arena. Also, Tsuruda and Page (2009) most likely used higher light intensities, since from the third light intensity onwards, all of their bees seem to have reached minimum walking times, while the walking times of our pollen and nectar foragers constantly decreased up to the highest light intensity. We therefore assume that the bees tested by Tsuruda and Page (2009) had already reached their maximum light responsiveness or highest walking speed in their run toward the third light intensity. Unfortunately, Tsuruda and Page (2009) did not measure the walking speed of their bees in the dark arena. This does not allow us to compare the locomotor behavior of their bees in the dark with that of our bees in the dark. Our findings do not only support the response threshold theory but imply that different groups of bees have different basic sensory response thresholds for light, which becomes an important tool in studying the mechanisms regulating basic sensory responsiveness.

## Conclusion

Taken together, our findings imply that responsiveness of bees to light is modulated by octopamine and tyramine. Octopamine treatment decreased light responsiveness, while tyramine treatment increased it. We therefore suggest that octopamine and tyramine have antagonistic functions in the evaluation of light stimuli, although both amines have a similar function in honey bee sucrose responsiveness. Pollen foragers displayed a lower responsiveness to light than nectar foragers. The lower responsiveness to light of pollen foragers is related to their higher octopamine titers in the optic lobes compared to nectar foragers but is independent of octopamine receptor expression. To what extent the differences in phototaxis of pollen and nectar foragers are causally related to division of foraging labor cannot be stated at this point. Future experiments employing techniques to knock down individual octopamine receptors or inhibit octopamine synthesis will help to elucidate the function of octopamine and individual octopamine receptors in regulating sensory responses and ultimately division of labor.

### Conflict of interest statement

The authors declare that the research was conducted in the absence of any commercial or financial relationships that could be construed as a potential conflict of interest.
